# Charting the evidence for climate change impacts on the global spread of malaria and dengue and adaptive responses: a scoping review of reviews

**DOI:** 10.1186/s12992-021-00793-2

**Published:** 2022-01-03

**Authors:** Manisha A. Kulkarni, Claudia Duguay, Katarina Ost

**Affiliations:** School of Epidemiology and Public Health, University of Ottawa, Ottawa, Canada

**Keywords:** Climate change, Malaria, Dengue, Adaptation, Vector-borne disease

## Abstract

**Background:**

Climate change is expected to alter the global footprint of many infectious diseases, particularly vector-borne diseases such as malaria and dengue. Knowledge of the range and geographical context of expected climate change impacts on disease transmission and spread, combined with knowledge of effective adaptation strategies and responses, can help to identify gaps and best practices to mitigate future health impacts. To investigate the types of evidence for impacts of climate change on two major mosquito-borne diseases of global health importance, malaria and dengue, and to identify the range of relevant policy responses and adaptation strategies that have been devised, we performed a scoping review of published review literature. Three electronic databases (PubMed, Scopus and Epistemonikos) were systematically searched for relevant published reviews. Inclusion criteria were: reviews with a systematic search, from 2007 to 2020, in English or French, that addressed climate change impacts and/or adaptation strategies related to malaria and/or dengue. Data extracted included: characteristics of the article, type of review, disease(s) of focus, geographic focus, and nature of the evidence. The evidence was summarized to identify and compare regional evidence for climate change impacts and adaptation measures.

**Results:**

A total of 32 reviews met the inclusion criteria. Evidence for the impacts of climate change (including climate variability) on dengue was greatest in the Southeast Asian region, while evidence for the impacts of climate change on malaria was greatest in the African region, particularly in highland areas. Few reviews explicitly addressed the implementation of adaptation strategies to address climate change-driven disease transmission, however suggested strategies included enhanced surveillance, early warning systems, predictive models and enhanced vector control.

**Conclusions:**

There is strong evidence for the impacts of climate change, including climate variability, on the transmission and future spread of malaria and dengue, two of the most globally important vector-borne diseases. Further efforts are needed to develop multi-sectoral climate change adaptation strategies to enhance the capacity and resilience of health systems and communities, especially in regions with predicted climatic suitability for future emergence and re-emergence of malaria and dengue. This scoping review may serve as a useful precursor to inform future systematic reviews of the primary literature.

**Supplementary Information:**

The online version contains supplementary material available at 10.1186/s12992-021-00793-2.

## Background

Climate change refers to the long-term change in the state of the climate [1]. Climate change influences a wide range of environmental factors, resulting in a rise in temperature, precipitation, sea level, ocean acidification and extreme weather events such as heat waves, floods, and storms. The Intergovernmental Panel on Climate Change (IPCC) has stated that human activities have already caused approximately 1.1 °C of global warming since the pre-industrial period, with each of the last four decades being successively warmer than any decade that preceded it since 1850 [2]. Moreover, the IPCC Sixth Assessment Report predicts that “global warming of 1.5°C and 2°C will be exceeded during the 21st century unless deep reductions in carbon dioxide (CO_2_) and other greenhouse gas emissions occur in the coming decades” [2]. The health impacts of climate change are wide-ranging and exacerbated by poverty and existing health inequities. Populations at disproportionately higher risk of adverse consequences from climate change impacts include those living in Arctic ecosystems, dryland regions, small island developing states, and Least Developed Countries [3].

Current evidence suggests that climate change and climate variability have a direct influence on the epidemiology of vector-borne diseases [1, 4]. Malaria and dengue fever are among the most important vector-borne diseases worldwide, with the highest disease burden occurring in tropical and subtropical regions. Malaria is a parasitic disease transmitted by *Anopheles* species mosquitoes; in 2019, there were an estimated 229 million cases of malaria and 409,000 deaths, with the majority of the burden of disease concentrated in sub-Saharan Africa [5]. Dengue is a mosquito-borne virus transmitted by *Aedes* species mosquitoes, which causes an estimated 100 million human cases and 10,000 deaths per year in over 125 countries [6–8]. The potential for climate-driven expansion and/or shifts in areas at risk of disease transmission for malaria and dengue has been demonstrated by numerous studies, ranging from laboratory studies on disease vector species response to temperature and environmental factors, modelling studies employing global climate change projections, and population-based studies in disease endemic regions, with some evidence for future emergence or re-emergence of transmission zones in more temperate regions and at higher altitudes [1, 3, 9]. For malaria, studies predict a northward shift of the malaria-epidemic belt in North America, Europe, and Asia, with anticipated increases in transmission suitability in tropical highland regions, including mountainous regions of sub-Saharan Africa, Latin America, the Western Pacific and the Eastern Mediterranean [4, 10]. A northward shift of the dengue-epidemic belt in parts of Europe and northern USA has also been predicted, with projected changes in dengue transmission in lowland areas of the Western Pacific and Eastern Mediterranean regions [4, 10]. In sub-Saharan Africa, there is concern that warming temperatures may result in a shift in disease burden from malaria to dengue and other arboviruses, due to differential changes in environmental suitability for the respective vector species [11]. Changes in the geographic range and intensity of malaria and dengue transmission under future climate change conditions, including spread into regions with immunologically naïve populations, may greatly increase the global population living in areas at risk and as a consequence the global disease burden attributable to these vector-borne diseases [10, 12].

Climatic factors, such as temperature and rainfall, are intricately linked to the biology and transmission of vector-borne diseases. According to the IPCC, global climatic changes, which include but are not limited to changes in temperature and rainfall, have altered the distribution of disease vectors and the risk of vector-borne diseases [1, 6, 13], and will continue to do so in future decades [8, 11], with the largest impacts experienced by populations living in resource-poor settings [10, 11]. For both malaria and dengue, which are transmitted by different genera of mosquitoes with different ecological niches, rainfall may contribute to the proliferation of mosquito breeding sites or conversely their ‘washing-out’ during heavy rainfall events, while temperature affects the development of the mosquito from egg to larva to adult, as well as the development of the pathogen within the mosquito’s body (the extrinsic incubation period) [12]. Mosquito biting rates and gonotrophic processes, which both affect the frequency at which a female mosquito feeds on a human host, are also temperature-dependent; thus increases in temperature can dramatically increase rates of disease transmission [14]. It is important to note that the relationships between climatic factors and disease transmission are complex and multi-faceted, with mosquito growth, survival and disease transmission potential restricted above or below certain temperature thresholds; optimum ranges for climatic suitability also vary according to vector species, pathogen and region, with disease transmission further influenced by other social and ecological factors.

The risk of spread of malaria and/or dengue in a given area depends on its receptivity, i.e. “the degree to which an ecosystem in a given area at a given time allows for pathogen transmission from a human to another human through a vector mosquito” [15]. The vectorial capacity of the mosquito population, susceptibility of the human population to infection and the strength of the health system, are all important considerations, in addition to local ecological and climatic factors. In areas at the fringes of current transmission, it is also important to consider the vulnerability of an area, which can be defined as the frequency of influx of infected individuals or groups and/or infective mosquitoes, i.e. importation risk [15].

While climate change mitigation efforts are critical to reduce CO_2_ emissions and the magnitude of future climate change impacts, there is increased focus on adaptation measures, which encompass a range of shorter-term actions to counter the impacts of climate change [16]. Adaptation strategies aim to build health system resilience to prepare for health impacts of extreme weather events, and reduce health effects of heat, among other national and sub-national actions by governments and communities [16]. Given the commitments that countries have made to the Paris Agreement and the Sustainable Development Goals (SDGs), and the growing global evidence base for climate change impacts on disease transmission and spread, countries have started to develop and adopt policy responses as part of national climate change adaptation plans [17]. Knowledge of the range and geographical context of the expected impacts of climate change on malaria and dengue, and of the associated adaptive responses, can help to inform best practices to mitigate public health impacts of climate-driven disease spread.

A recent bibliometric analysis of the climate change and health-related literature revealed that malaria and dengue were among the most-studied climate-related infectious diseases [9]. A steady increase in the number of published studies was noted since 2007, in line with the recognition of climate change impacts on health by the IPCC 4th Assessment Report [18], with further increasing publication trends following the IPCC 5th Assessment Report [1] and the 2015 Lancet Commission on Climate Change and Health [19]. In addition to the proliferation of primary research papers, reviews that specifically address aspects of malaria and/or dengue in the context of climate change have increased in number in recent years. These reviews have typically focused on the impacts of climate change, or to a lesser extent on specific interventions or policy responses, in specific countries or other geographical contexts (e.g. Asia). To our knowledge, a comprehensive review of the global impacts and adaptation strategies related to climate change and malaria and/or dengue has not been conducted. Such a review would help to synthesize the evidence and identify evidence gaps related to climate-driven disease spread for malaria and dengue, as well as highlight geographical contexts with the greatest impacts and/or progress in developing policy responses for climate change adaptation.

As a first step in developing a comprehensive review (i.e. systematic review of the primary literature), scoping reviews can provide a useful means to identify the types of available evidence in a given field and confirm the relevance of potential research questions [20]. We therefore conducted a scoping review that aimed to broadly identify and map the available evidence for impacts of climate change on the transmission and spread of two major mosquito-borne diseases of global health importance, malaria or dengue, and to identify the range of relevant policy responses and adaptation strategies that have been devised. Given the considerable volume of primary research on this topic, we performed a scoping review of published review literature guided by the methods for scoping reviews of published systematically-conducted review studies [21] and the PRISMA extension for scoping reviews (PRISMA-ScR) [22] (see Methods for details).

This scoping review aims to address the question, “What types of evidence exist for the impact of climate change on the transmission and spread of malaria and dengue, and what types of policy responses (adaptation measures) have been devised?”

In addition, this scoping review aims to address two sub-questions:

1. In what geographical contexts is the evidence for climate change impacts on malaria and/or dengue transmission and spread the greatest?
2. In what geographical contexts have relevant policy responses (adaptation measures) been devised to address climate-driven impacts on malaria and/or dengue transmission and spread?

## Results

### Characteristics of reviewed articles

**Fig. 1:**
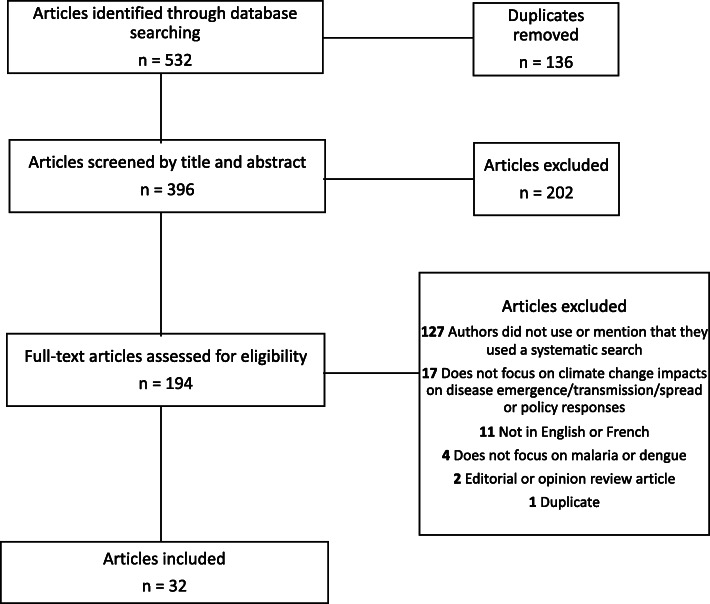
PRIMSA flow chart

Our searches yielded a total of 532 articles, after the removal of 136 duplicate records (Fig. 1). We removed 202 studies upon screening of article titles and abstracts, which excluded those that did not specifically address either climate change impacts or policy responses and adaptation measures related to either malaria or dengue. Upon full-text review, we removed 162 articles, the majority (*n* = 127) of which did not mention or use a systematic search strategy.

Characteristics of the articles included in the review are presented in Table 1. Of the 32 included reviews, 63% (*n* = 20) were systematic reviews, one of which included a meta-analysis; in addition, 16% (*n* = 5) were scoping reviews, 9% (*n* = 3) were narrative reviews, 9% (*n* = 3) were critical reviews, and 3% (*n* = 1) was a realist review (Table 1) [4, 14–17, 23–49].

**Table 1:** Characteristics of the articles included in the review

Citation	Objective	Type of review	Databases searched	Years included in review	Geographic focus	Disease of interest	Degree of disease focus	Addresses climate change impacts	Addresses adaptation measures
*African Region*									
(Abiodun et al., 2020) [23]	To establish the major factor responsible for the recent malaria resurgence in South Africa between 2015 and 2018.	Scoping review	CINAHL, PubMed, Science Direct, SCOPUS	2015–2018	South Africa	Malaria	Primary	Y	N
(Chersich and Wright, 2019) [16]	To assess progress with climate change adaptation in the health sector in South Africa.	Systematic review	PubMed (Medline, Web of Science	No time limits	South Africa	Malaria	Secondary	N	Y
(Giesen et al., 2020) [24]	To assess the effects of climate change in the epidemiology of the most prevalent mosquito borne diseases and their vectors in Africa.	Systematic review	PubMed, Scopus, Embase, CENTRAL	2004–2018	African Region	Both	Secondary	Y	N
(Mabaso and Ndlovu, 2012) [25]	To obtain a better understanding of existing research evidence towards the development of climate-driven malaria early warning systems (MEWS) in order to identify challenges and opportunities for future research.	Critical review	PubMed	1990–2009	African Region	Malaria	Primary	Y	Y
*Americas*									
(López et al., 2018) [26]	To analyze the distribution and abundance of publications on vector-borne diseases associated with climate variability in South America, identify those works that conducted a geographic analysis and detect the countries where outbreaks occurred and the climate variables with which they were associated.	Systematic review	Scielo, PubMed, Lilacs, Google Scholar, Scopus	1970–2016	South America	Both	Secondary	Y	N
(Matysiak and Roess, 2017) [27]	To examine the interrelationship between climatic, ecological, social, and cultural factors on dengue transmission in Puerto Rico and to draw lessons for Zika response.	Systematic review	MEDLINE, PUBMED, SCOPUS, CINAHL	2001–2015	Puerto Rico	Dengue	Primary	Y	N
*Eastern Mediterranean*									
(Babaie et al., 2018) [28]	This study aimed to review the effects of climate change on malaria in Iran.	Systematic review	Cochrane, PubMed, ScienceDirect, SID, Magiran	2007–2017	Iran	Malaria	Primary	Y	N
(Khan et al., 2018) [29]	To conduct a comprehensive compilation of dengue cases from published data and known records in the country, and use a modeling framework to understand dengue prevalence and risk.	Systematic review and meta-analysis	Science Citation Index, SciSearch, Journal Citation Reports, Medline, SCOPUS, EMBASE, Google Scholar, and others	1994–2014	Pakistan	Dengue	Primary	Y	N
(Ahmed et al., 2016) [30]	To critically review scientific studies to assess the need for water conservation, risk management, and the development of mitigation measures to cope with the water-related impacts of climate change on agriculture and subsequently on public health, particularly from the Pakistani perspective.	Critical review	Google Scholar, Web of Science, Scopus, and Science Direct	1980–2016	Pakistan	Both	Secondary	Y	N
(Ahmed et al., 2019) [31]	To assess the existing conventional and novel eradication methods and techniques, which are being used in different countries of the world to eradicate or control vectors and diseases transmitted by such vectors, and to identify missing gaps in the management of vectors, especially malaria and dengue fever, and approaches to manage increases in temperature due to a changing climate	Critical review	ISI Web of Knowledge, Science Direct, Scopus and Google Scholar	1990–2019	Pakistan	Both	Secondary	Y	Y
*European*									
(Brugueras et al., 2020) [32]	To identify and analyze the existing literature on the transmission of mosquito-borne diseases and those factors potentially affecting their transmission risk of them in six southern European countries with similar environmental conditions: Croatia, France, Greece, Italy, Portugal and Spain.	Systematic review	PubMed, Embase, Scopus, Web of Science, AHL Regional Portal	2000–2017	Croatia, France, Greece, Italy, Portugal and Spain	Both	Secondary	Y	N
(Fischer et al., 2020) [15]	To assess the impact of rising temperature on the receptivity to malaria transmission in Europe and to provide an evidence base for the critical appraisal of the current state of knowledge on which health care guidelines and prevention efforts rely.	Systematic review	Embase, Medline, Cochrane Library, Scopus	Before Oct 2019	Europe	Malaria	Primary	Y	N
(Medlock and Leach, 2015) [33]	To summarise the risks posed by vector-borne diseases in the present and the future from a UK perspective, and assess the likely effects of climate change and, where appropriate, climate-change adaptation strategies on vector-borne disease risk in the UK.	Systematic review	PubMed, Google Scholar, Web of Science	Before Oct 2014	UK	Both	Secondary	Y	Y
*South-East Asian*									
(Chua et al., 2019) [34]	To map out the extent of climate change and health research done in the country in order to complement the agenda-setting process and guide in identifying more specific research topics for the Philippine National Unified Health Research Agenda 2017–2022 under the Health Resiliency section.	Scoping review	PubMed/MEDLINE, Embase, Web of Science, HERDIN	1980–2017	Philippines	Both	Secondary	Y	Y
(Dhimal et al., 2015) [35]	To review the available literature on VBDs and climate change to allow for an assessment of the likely impacts of climate change on the changing spatiotemporal distribution of VBDs in Nepal.	Systematic review	PubMed, Web of Science	Before Dec 2014	Nepal	Both	Secondary	Y	N
(Hii et al., 2016) [14]	To review the current status of scientific studies in climate and dengue and the prospect or challenges of such research on a climate-based dengue early warning system in a dengue endemic country, taking Malaysia as a case study.	Systematic review	PubMed, Scopus, EBSCO, Web of Science, WHOLIS, WHO IMSEAR	1990–2015	Malaysia	Dengue	Primary	Y	N
*Western Pacific*									
(Bai et al., 2013) [36]	To summarize what is known about the impact of climate change on the incidence and prevalence of malaria, dengue fever and Japanese encephalitis in China and to provide important information and direction for adaptation policy making.	Scoping review	PubMed, Google Scholar and China Hospital Knowledge Database (CHKD)	Before 2012	China	Both	Secondary	Y	Y
(Li et al., 2018) [37]	To summarize empirical evidences in China on the impact of climate change on dengue fever and to review the related DF incidence models and their findings on how changes in weather factors may impact DF occurrences in China.	Systematic review	Google Scholar, Web of Science/Knowledge, PubMed, Baidu Scholar, and CNKI	1980–2017	China	Dengue	Primary	Y	N
(Filho et al., 2019) [38]	To explore the associations between climate change and human health on the one hand, and outline some of the health care challenges posed by a changing climate on the other, including the emergence of climate-sensitive infectious diseases.	Narrative	PubMed and Google Scholar, and scientific reports (IPCC, NASA, ECDC, or WHO)	2004–2019	Western Pacific Region	Dengue	Secondary	Y	Y
(Yi et al., 2019) [39]	To examine the relationship between climate variability and infectious disease transmission in China in the new millennium.	Systematic review	Web of Science, PubMed, CNKI	2000–2018	Western Pacific Region (China)	Both	Secondary	Y	Y
(Banu et al., 2011) [40]	To review the scientific evidence about the impact of climate change and socioenvironmental factors on dengue transmission, particularly in the Asia-Pacific region.	Scoping review	PubMed, ISI web of Knowledge and Google Scholar	1990–2009	Asia-Pacific (South-East Asia Region AND Western Pacific Region)	Dengue	Primary	Y	Y
*Global*									
(Akter et al., 2017) [42]	To assess the epidemiological evidence on the joint effects of climate variability and socioecological factors on dengue transmission	Systematic review	PubMed, Web of Science and Scopus	1993–2015	Global	Dengue	Primary	Y	N
(Andersen and Davis, 2017) [43]	To gather available literature describing changes in the epidemiology of tick- and mosquito-borne diseases that cause cutaneous manifestations, which may be associated with climate change.	Scoping review	PubMed	1984–2016	Global	Both	Secondary	Y	N
(Bardosh et al., 2017) [17]	To identify community-based interventions for VBDs with the goal of relating past approaches and lessons learnt to the context of future global change. To situate the existing community-based VBD intervention literature within the context of global change processes, the broader socioecological systems theory literature, social science knowledge and concepts of vulnerability and adaptation.	Realist review	PubMed and Google Scholar	1990–2015	Global	Both	Secondary	Y	Y
(Cella et al., 2019) [44]	To elucidate the important aspects described in the literature on the influence of climate change in the distribution and transmission of malaria.	Narrative	PubMed and SciELO Virtual Library	1994 to 2018	Global	Malaria	Primary	Y	N
(Naish et al., 2014) [41]	To review epidemiological evidence on the relationship between climate and dengue with a focus on quantitative methods for assessing the potential impacts of climate change on global dengue transmission.	Systematic Review	PubMed, Scopus, ScienceDirect, ProQuest, and Web of Science	January 1991 through October 2012	Global	Dengue	Primary	Y	N
(Swynghedauw, 2009) [45]	To review the medical consequences of global warming.	Systematic review	PubMed	2000–2007	Global	Both	Secondary	Y	N
(Watts et al., 2021) [4]	To report the findings and consensus of the Lancet Countdown, an international collaboration established to provide an independent, global monitoring system dedicated to tracking the emerging health profile of the changing climate.	Narrative	N/A	Up to 2020	Global	Both	Secondary	Y	Y
(Xu et al., 2012) [47]	To review the literature regarding the relationship between ambient temperature and children’s health and to propose future research directions.	Systematic review	PubMed, ProQuest, ScienceDirect, Scopus and Web of Science	Up to Feb 2012	Global	Malaria	Secondary	Y	N
(Xu et al., 2020) [46]	To review available information on the projection of dengue in the future under climate change scenarios.	Systematic review	PubMed, ProQuest, ScienceDirect, Scopus and Web of Science	Up to June 2019	Global	Dengue	Primary	Y	N
(Yu et al., 2015) [48]	To examine both key findings and methodological issues in projecting future impacts of climate change on malaria transmission.	Critical review	MEDLINE, Web of Science, and PubMed	Up to Nov 2012	Global	Malaria	Primary	Y	N
(Zhang et al., 2008) [49]	To summarize what has been done in examining the relationship between climate change and vector-borne diseases worldwide and to give suggestions for future research directions by noting limitations in previous published work.	Systematic review	PubMed	1984–2008	Global	Both	Secondary	Y	N

**Fig. 2:**
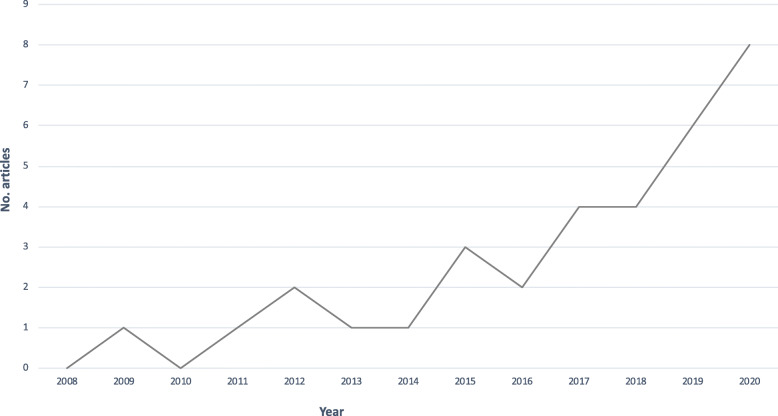
Number of eligible review articles over time

The number of review articles increased over time, with the greatest increase noted from 2015 onwards (Fig. 2). This timing follows the release of the IPCC Fifth Assessment Report in 2014 [1] and publication of the 2015 Lancet Commission on Climate and Health [19].

**Fig. 3:**
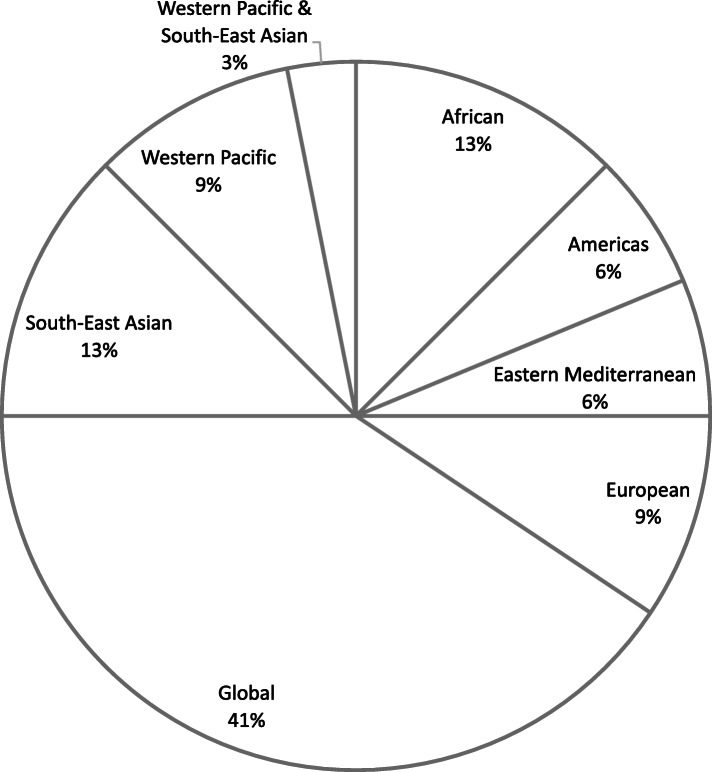
Distribution of eligible review articles by geographic region

94% (*n* = 30) of the 32 review articles addressed climate change impacts on malaria and/or dengue, while 34% (*n* = 11) addressed policy responses or adaptation measures related to dengue and/or malaria. The majority of review articles (41%, *n* = 13) included studies from all geographic regions, while 13% (*n* = 4) included studies from the Southeast Asian region and 13% (*n* = 4) from the African region. A smaller number of articles reviewed the evidence from the Western Pacific (9%, *n* = 3) and European regions (9%, *n* = 3), while the Eastern Mediterranean and Americas accounted for 6% (*n* = 2) each. One article, accounting for 3% of all articles, reviewed studies from the Asia-Pacific region and was classified as Western Pacific & Southeast Asian (Fig. 3).

**Fig. 4:**
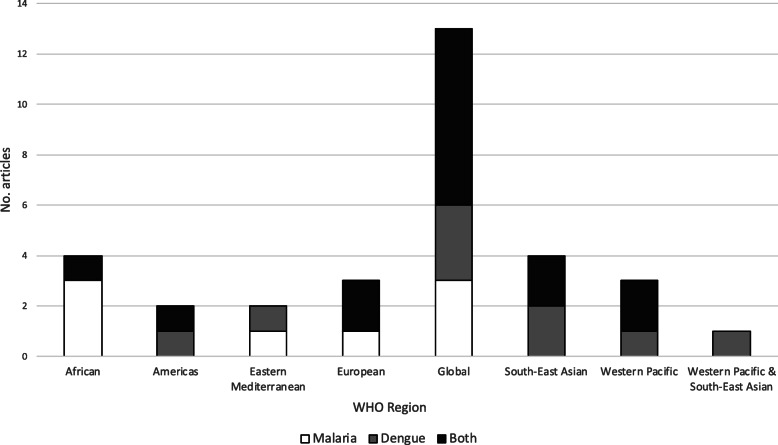
Number of eligible review articles by region and disease(s) of focus

The majority of review articles (47%, *n* = 15) addressed both malaria and dengue, typically amongst a broader range of climate-sensitive infectious diseases. Twenty-eight percent (*n* = 9) of the eligible review articles had a focus on dengue, while 25% (*n* = 8) had a focus on malaria. Both malaria and dengue were addressed to some extent in reviews in each of the geographic regions, however malaria was the main focus in the African region and dengue was the main focus in the Southeast Asian region (Fig. 4).

The evidence for climate change impacts and adaptation measures related to dengue and malaria transmission and spread in different geographic regions is summarized in Table 2, with further detail on the results from individual review articles in the Supplemental Materials (Table S1). A narrative synthesis of results is presented below.

**Table 2:** Summary of key findings on climate-disease associations, climate change impacts on disease and adaptation strategies by geographic region and relationship

**Climate-disease associations:**	
ENSO variability associated with malaria epidemics: African region [25]	
Increased rainfall and temperature associated with malaria epidemics: South Africa [23], East African highlands [25], South America [26], Iran [28], Nepal [35]	
Temperature limits malaria vector occurrence and abundance: Europe [32]	
Humidity associated with malaria transmission: Pakistan [30, 31], Iran [28]	
ENSO variability and dengue incidence: South America [26], Asia-Pacific [38, 40]	
Variations in temperature, precipitation, and dengue incidence: Peurto Rico [27], Pakistan [29–31], China [36, 37, 39], Malaysia [14]	
**Climate change impacts on disease:**	
Increased malaria transmission suitability at higher altitudes: African highlands, Latin America, Southeast Asia [4, 17, 24], Western Pacific [48], Nepal [35]	
Latitudinal expansion of Anopheles mosquitoes: Spain, France, Italy, Greece, Central and Eastern Europe [15], China [39]	
Increasing *Plasmodium vivax* malaria transmission suitability: UK [33], Europe [15]	
Reduced malaria transmission suitability due to extreme temperatures: Philippines [34]	
Expansion of *Aedes* mosquitoes to higher altitudes: Nepal [35]	
Increased dengue transmission suitability: African region [24], China [37]	
Latitudinal expansion of *Aedes* mosquitoes: Europe [32], South America [26], UK [33]	
**Adaptation strategies:**	
Enhanced surveillance for non-native vectors: UK [33]	
Malaria early warning systems (MEWS): South Africa [16], African region [25], Philippines [34]	
Environmental management and enhanced vector control: UK [33], Pacific region [38]	
Early warning systems for dengue outbreaks: Philippines [34]	
Predictive models of future climate change impacts: UK [33]	
National adaptation planning and health systems strengthening: Nepal [35]	

### Climate-dengue associations

Positive associations between short-term variations in meteorological factors and dengue incidence were frequently reported, although the nature of effects often varied at different regional and sub-national scales [41]. Precipitation, temperature and humidity were all associated with dengue incidence in the Americas, including an effect of El Niño on *Aedes* mosquito populations [26]. In Puerto Rico, the effect of temperature on dengue incidence was highest in the country’s mountainous area, while the effect of precipitation was greatest in the hot and dry coastal region [27]. Similarly, in Pakistan, the seasonal transmission pattern of dengue occurs after the monsoon when higher rainfall, combined with optimum temperature and humidity, provides a conducive environment for *Aedes* mosquitoes [29–31]. In China and other countries in the Asia-Pacific, temperature, precipitation, humidity and air pressure were considered as major weather factors for dengue fever (DF) transmission by many studies, including in Zhongshan City, Guangdong Province, and in Guangzhou City [37].

However, it is important to note that increases in temperature and precipitation do not necessarily translate to higher disease incidence - regions that are currently within optimal temperature ranges for dengue transmission may experience a decrease in disease incidence with increasing temperature [37], while intense rainfall may reduce dengue incidence through elimination of larvae from overflowing containers and other breeding sites, as observed in South America, Thailand, Indonesia and Taiwan [26, 40]. Indeed, in Malaysia a positive relationship between minimum temperature and dengue cases was observed with the highest risk of dengue cases observed from 21 to 26 °C at a lag of 1–8 weeks [14]. Furthermore, several non-climatic factors, such environmental and socioeconomic changes, population movements, and population immunity, may potentially confound assessments of the climate-dengue relationship [35, 40], leading to inconsistencies in the empirical evidence linking dengue fever to climate change across different geographical locations.

### Climate change impacts on dengue

According to Watts et al., from 1950 to 2018, the global climate suitability for the transmission of dengue increased by almost 10% for *Aedes aegypti* and 15% for *Ae. albopictus*, the primary vectors of dengue virus [4]. Ongoing climate change is anticipated to further extend the latitudinal range *Ae. aegypti* mosquitoes, increasing the population at risk of dengue in several African countries in Southern and Central Africa [24]. Similar increases in climatically suitable areas for the establishment of *Ae. albopictus* are anticipated in western, central and eastern Europe, including in southeast England, with increasing risk for dengue transmission around Mediterranean and Adriatic coasts towards the end of twenty-first century [32, 33]. In China, there has been a trend of expanded geographical region for dengue infections, from South to North China in line with warming temperatures [39] and expansion of the geographic range of *Ae. albopictus* [36]. In Nepal, Dhimal et al. conclude that climate change can intensify the risk of dengue epidemics in the mountain regions of the country, where *Ae. aegypti* vectors have been rapidly expanding, if other non-climatic drivers remain constant [35].

### Climate-malaria associations

Malaria prevalence and epidemic resurgence has been significantly associated with temperature and rainfall, and to El Niño-Southern Oscillation (ENSO) events in South Africa, the East African highlands, and Madagascar [23–25]. Similar to dengue, the role of epidemiological, socio-economic and environmental factors in driving malaria transmission has been noted in many geographical areas [23]. Precipitation and/or temperature have been positively associated with malaria incidence and vector population in Americas [26], Europe [32], Iran [28], Pakistan [30, 31], China [36] and Nepal [35].

### Climate change impacts on malaria

There is strong consensus among the reviewed studies that climate change is expected to increase malaria transmission at higher altitudes in the highlands of Africa, parts of Latin America and Southeast Asia, and in other regions at the margin of current distributions depending on demographic, socio-economic and ecological factors [4, 17, 24, 44, 45, 48]. In China, in the absence of preventive measures, climate change is anticipated to increase the geographical range of local malaria vectors and the incidence of malaria in some regions [39]. In Europe, studies have predicted a northward spread of *Anopheles* mosquitoes and an extension of seasonality, enabling malaria transmission for up to 6 months per year in the years 2051–2080, particularly in Southern and South-Eastern European [15], while in the UK, southern Great Britain is predicted to be climatically suitable for *Plasmodium vivax* malaria transmission 2 months of the year by 2030 and for 4 months in parts of southeast England; by 2080, southern Scotland will be climatically suitable for malaria transmission for 2 months per years, with 4 months of the year conducive to malaria transmission in southern Great Britain [33]. In some areas that currently sustain year-round malaria transmission, climate change may result in a contraction of the malaria transmission season or geographic range. For example, in the Philippines, model projections show an overall reduction in the climate suitability for Anopheles due to heat stress, causing large areas to exceed thresholds for of malaria vectors (> 40 °C) [34].

### Adaptation strategies to address climate-driven malaria and dengue transmission and spread

The majority of reviewed studies recommend further emphasis on developing predictive models and early warning systems (EWS) to enhance outbreak preparedness and response, in addition to strengthening the capacity of surveillance and control systems. Enhanced epidemic prediction capability can allow for early intervention and more effective resource allocation. Importantly, predictive models and EWS should aim to integrate climate and meteorological factors alongside non-climate factors, such as socioeconomic variations, land use changes (including urbanization) and population growth, to more accurately predict disease emergence and/or spread [4, 14, 16, 17, 25, 32, 35–37, 39, 44, 46]. As noted by Hii et al., the development of models will require that governments make human case data publicly available for research purposes and that they support synchronized efforts across national disease surveillance systems and meteorological departments to develop climate-based disease forecasting systems [14]. As noted by several studies, vector-borne disease monitoring, surveillance and research should be strengthened, including in areas where risk of vector-borne diseases is not yet determined, to assess the impacts of climate change on the observed transmission and distribution of vector-borne diseases in new areas. Well-designed long-term local studies are needed to provide the relevant information to develop locally-relevant models and responses [35, 37]. For example, Li et al. highlights the need to promote more advanced research on the relationship between extreme weather events and dengue fever to develop regional-specific models for the high-risk regions of dengue fever in south China. Ultimately, enhancing interdisciplinary collaboration between climate studies and health services, and enhancing public health education, are both future priorities. Bai et al. points to the additional need to focus adaptation strategies and policies on vulnerable communities while strengthening the capacity of public health system to adapt to climate change [36]. As noted by several studies, new vector control strategies, such as wetland management and integrated vector management (IVM), will be needed, despite the challenges of funding and inter-sectoral cooperation [17, 30, 33].

Importantly, Bardosh et al. highlight the need to recognize that the myriad global changes, including climate change, land use, agriculture, dams, irrigation, urbanization, economic development, population movement, conflict, socio-political shifts, biological change, drug resistance, etc., do not occur in isolation [17]. As such, predictions of expanded disease transmission should take into consideration current control initiatives, economic development trends and the future adaptation measures implemented by local populations and public health agencies. Importantly, successful adaptation and response will require interdisciplinary collaboration between meteorologists, biologists, climate scientists, social scientists, and epidemiologists, as well as partnerships with local communities to integrate local knowledge [17, 41].

As noted by Watts et al., adaptation planning and risk management is essential across all levels of government, with national strategies linked to subnational and local implementation [4]. According to the 2020 Lancet Countdown Report, 50% of 101 countries surveyed had developed national health and climate change strategies or plans while 48% had assessed national vulnerability and adaptation for health [4]. However, funding was highlighted as a key barrier to implementation of these strategies, with only 9% of countries reporting to have the funds to fully implement their plans. Encouragingly, the number of countries reporting that their meteorological services provide climate information to the health sector has grown in recent years.

## Discussion

This scoping review of the published review literature identified strong evidence for climate-disease relationships and the impacts of climate change on the transmission and future spread of malaria and dengue, two of the most globally important vector-borne diseases. A large number of the review articles that were included in our scoping review identified studies examining associations between weather and climate factors (e.g. temperature, rainfall, humidity) and disease incidence or vector populations, while fewer reviews identified studies examining observed or predicted impacts of climate changes on disease incidence or risk. There was general consensus that the areas at greatest risk of climate-driven spread and emergence include regions at the margins of current disease transmission, due to the latitudinal and altitudinal expansion of disease vectors or the importation of pathogens into regions where competent vector populations are present. However, importantly, many studies highlighted that local associations between climate and meteorological factors and disease incidence may vary depending on other confounding factors, including socioeconomic, demographic, and land use changes, highlighting the importance of accounting for these variables in climate change projections.

### Limitations

Despite the breadth and wide geographic coverage of the evidence reviewed, this scoping review is subject to some limitations. Given the aim to review evidence from existing review articles, rather than primary studies, it is likely that more recent evidence of climate change impacts and/or policy responses and adaptation measures from some geographic regions was not captured in the reviews published to date. However, the review approached used herein is useful for identifying strengths and gaps in the evidence across different geographic regions and can serve as a precursor to a systematic review of the primary literature. Future reviews could consider narrower search terms or inclusion criteria to focus on studies investigating climate change impacts and adaptation measures, and to exclude the wide range of studies that focus on climate/weather-disease associations without explicitly addressing the impacts of climate change. While the majority of studies were systematic reviews, we did not undertake a quality assessment of the included reviews given the scoping review methodology. This may have resulted in the inclusion of lower quality reviews in our evidence mapping, however all included studies used a systematic search strategy, increasing the rigour of our approach, and there was strong agreement of findings across different review types.

## Conclusions

Climate change is predicted to alter the risk of mosquito-borne diseases, increasing transmission suitability for malaria and dengue in temperate regions and expanding the global population at risk for these disease. Adaptive strategies to anticipate and respond to the climate-driven spread of malaria and dengue are urgently needed, however studies focusing on adaptation strategies are sparse in comparison to those examining climate change impacts. Further research on climate change impacts and adaptation strategies for mosquito-borne diseases, and further evidence synthesis, is needed to inform effective policy responses that are tailored to local contexts.

## Methods

### Protocol and registration

Scoping reviews aim to comprehensively and systematically map the published literature on a broad topic [20]. Scoping reviews do not require quality assessment, yet they provide a rigorous and methodical approach to examining the extent and characteristics of research in a particular field. We performed a scoping review using a pre-defined protocol, guided by the ﻿methods for scoping reviews of published systematically-conducted review studies [21] and the PRISMA extension for scoping reviews (PRISMA-ScR) [22]. The protocol for this review was registered on OSF Registries on December 11, 2020 (https://osf.io/bp47u).

### Eligibility criteria

The inclusion and exclusion criteria that were applied to assess the eligibility of articles are shown in Table 3. We chose to examine the literature since 2007 to capture the period following the release of the Intergovernmental Panel on Climate Change (IPCC) Fourth Assessment Report [18]. During this period, there was a noticeable increase in the number of published studies on climate-related infectious diseases [9]. We included reviews for which there was a systematic process in selecting studies to increase the rigour of our review, and focused on published articles in English or French.

**Table 3:** Criteria for inclusion and exclusion

Category	Inclusion	Exclusion
Concept	- Articles on climate or climate change impacts on disease emergence, transmission or spread AND/OR policy responses, interventions or climate change adaptation measures related to disease emergence, transmission or spread - Articles on malaria and/or dengue	- Articles on disease prevention and control interventions for malaria and/or dengue that are not explicitly related to climate change (e.g. RCTs) - Articles on climate impacts or interventions related to mosquito species with no mention of implications for malaria and/or dengue emergence, transmission or spread
Type of evidence sources	- Systematic reviews and reviews with some description of a systematic approach to study selection (e.g. systematic review OR scoping review OR scoping study OR rapid review OR critical review)	- Reviews without some description of a systematic approach to study selection (e.g. opinion piece OR editorial)
Language	- Articles in English or French	- Other languages
Timeframe	- Articles published in 2007 or later	- Articles published prior to 2007
Publication status	- Articles published or in press	- Pre-print articles (i.e. prior to peer review)

### Search strategy

A search was conducted on December 11, 2020 in the following databases: PubMed (MEDLINE), Scopus and Epistemonikos. The search included all types of reviews published since 2007 with no language restrictions. The literature search included terms specific to climate change and the diseases of interest. The terms related to climate change were derived from the search strategy previously employed in the recent bibliometric analysis on climate change and health-related literature by Sweileh (2020): “Climat* Change” OR “greenhouse effect” OR “changing climate” OR “global warming” OR “extreme weather” OR “climate variability” OR “greenhouse gas” OR “rising temperature”. Terms related to the specific diseases of interest were also included: “malaria” OR “dengue”. The search strategies for each database are shown in the Supplemental Materials (Table S2).

### Screening and study selection

All reviews were imported into Covidence (Veritas Health Innovation, Melbourne, Australia; available at www.covidence.org), a systematic review software, for the screening and management of the results of the search. Two reviewers independently screened article titles and abstracts to assess whether each article met the inclusion criteria, and any discrepancies were resolved by a third reviewer. Two reviewers then assessed all screened-in articles via full text review to confirm that the retained articles met all inclusion criteria, including a systematic search methodology. Any discrepancies were resolved by a third reviewer.

### Data extraction

We used a pre-specified data extraction form, developed using Covidence, to extract information on the characteristics of the article (title, author(s) and year) [2]; context of the article (objectives, type of review, disease(s) of focus, degree of focus on malaria and/or dengue [primary/secondary], and geographic focus); and [3] findings of the paper, i.e. nature of the evidence for climate change impacts on disease emergence, transmission or spread and/or policy responses, interventions or adaptations.

The type of review was specified as systematic review, scoping review, critical review, or other, based on the specified methodology of each article. The disease(s) of focus was specified as “malaria”, “dengue”, or “both”, while the degree of focus was specified as “primary” if malaria or dengue was the primary focus of the article, or “secondary” if the article addressed multiple diseases, of which malaria or dengue was among the diseases of interest. The geographic focus was specified as the country of focus or the World Health Organization (WHO) region if more than one country.

The findings of the paper pertaining to evidence for climate change impacts on disease emergence, transmission or spread was recorded as the nature of the evidence or the argument that was presented regarding the impact(s) of climate change (including rising temperatures, extreme weather, and/or climate variability) on malaria and/or dengue emergence, transmission or spread. This could include the magnitude of observed or predicted impacts on specific settings/populations, and/or how a specific climatic driver (e.g. temperature, precipitation) affects disease transmission through impacts on hosts, vectors, pathogens and/or the environment.

The findings of the paper pertaining to evidence for policy responses, interventions or adaptive measures to address climate change impacts on disease emergence, transmission or spread was recorded as the nature of the evidence or the argument that was presented regarding policy response, interventions and/or adaptation measures to mitigate climate change impacts on malaria and/or dengue emergence, transmission or spread. We noted whether the article mentioned that the policy response, intervention or adaptive strategy was part of a country’s Health National Adaptation Plan (H-NAP).

We summarized the evidence according to the disease of focus, nature of the evidence, and geographic focus in order to identify and compare regional evidence for climate change impacts and adaptation measures related to malaria and dengue emergence, transmission and spread.

## Supplementary Information

**Additional file 1.**

## Data Availability

All data generated or analysed during this study are included in this published article [and its supplementary information files].

## Abbreviations

IPCC: Intergovernmental Panel on Climate Change
ENSO: El Nino-Southern Oscillation
EWS: Early warning system
