# Supplementary material for: Charting the evidence for climate change impacts on the global spread of malaria and dengue and adaptive responses: a scoping review of reviews
Source: Global Health. 2022 Jan 3;18:1. doi: 10.1186/s12992-021-00793-2 (PMC8725488; doi:10.1186/s12992-021-00793-2)
Supplement: Supplementary file 1 — Additional file 1. [file 12992_2021_793_MOESM1_ESM.docx]

**Supplemental Materials – Charting the evidence for climate change impacts on the global spread of malaria and dengue and adaptive responses: a scoping review of reviews**

**Table S1.** Detailed summary of results pertaining to climate change impacts on malaria and dengue, and adaptation strategies to address climate-driven disease spread

| Malaria | No. articles | Results | Citations |
| --- | --- | --- | --- |
| Climate Impacts  Climate/weather-disease associations | 8 | In Africa, extreme climatic conditions are predisposing factors for malaria epidemics; there is a relationship between ENSO events and unusual climatic conditions that lead to malaria epidemics. In highland areas of Ethiopia and Rwanda, malaria epidemics were associated with increased rainfall and elevated minimum temperature. In South Africa, climatic conditions were found to be the major drivers of the resurgence of malaria, while the role of epidemiological, socio-economic and environmental factors was also noted.  In South America, most studies revealed a positive association between the number of malaria cases and/or different biological variables (vectors abundance, frequency, persistence and mortality, among others) and precipitation and/or temperature.  In Europe, the presence of appropriate vectors and climatic conditions, and high frequency of human movements from malaria endemic countries may favour the reintroduction of malaria in Greece and parts of Italy, Spain and Croatia, and the Camargue region of France; temperature was a key factor to be able to understand malaria vector abundance and expansion.  In Iran, malaria outbreaks are strongly correlated with precipitation and humidity with a significant relationship between malaria transmission and the minimum and maximum temperature in all of the studied regions.  In Pakistan, increases in heat and humidity are associated with an increase in vectors and malaria, while increase in droughts is associated with a decrease in vectors and malaria.  In China, in general there were significant positive relationships between malaria incidence and maximum temperature for both P. vivax and P. falciparum malaria, although associations varied across province of study; the impact of precipitation on malaria transmission is inconsistent across geographical locations in China.  In Nepal there has been a gradual shift in the Plasmodium parasite population from P. vivax to P. falciparum, possibly due to the rising temperature trends. Positive relationship between rainfall and malaria cases with a certain time lag has been observed in Nepal as well as a significantly positive correlation of malaria incidence with minimum as well as maximum temperatures and rainfall. | Mabaso and Ndlovu, 2012, Abiodun et al., 2020  López et al., 2018  Brugueras et al., 2020  Babaie et al., 2018  Ahmed et al., 2016 & 2019  Bai et al., 2013  Dhimal et al., 2015 |
| Climate change impacts on disease | 10 | Studies on malaria distribution have projected increased transmission at higher altitudes in the highlands of Africa, parts of Latin America and Southeast Asia. More recent studies show increases in areas other than the highlands, depending on demographic, socio-economic and ecological factors. Most studies reveal changes at the margin of current distributions, where non-immune populations present “endemically unstable areas”, which will shift patterns of endemic/epidemic conditions.  The IPCC predicts that in 2050, malaria may threaten some previously unexposed regions of South America, sub-Saharan Africa (SSA), and China, thus causing a 50% higher probability of malaria. Specific impacts of climate change on increasing malaria have been identified in Sub-Saharan Africa (East Africa, Ethiopia and African continent), Colombia, and southern China.  In 2015–19, suitability for malaria transmission in highland areas was 38·7% higher in the African region and 149·7% higher in the Western Pacific region compared with a 1950s baseline. Increasing temperatures are eroding the effect altitude has as a barrier to malaria transmission which has resulted in more favourable conditions in densely populated highland areas, as seen in Ethiopia. There have been significant increases in the environmental suitability for the transmission of falciparum malaria in highland areas of four of the five malaria endemic regions, with an increase of 38·7% in the African region and 149·7% in the Western Pacific region in 2015–19 compared with the 1950s baseline.  Malaria prevalence will be affected by CC in several African countries, specifically the East African highlands  The population at risk of malaria is projected to increase in East and South Africa, but decreasing in West Africa; there will be a northward expansion into West Asia and East Asia by 2030, 2050, and up to 2080  In Europe, increasing temperatures are predicted to result in a northward spread of the occurrence of Anopheles mosquitoes and an extension of seasonality, enabling malaria transmission for annual periods up to 6 months in the years 2051–2080, with increased risk in: Spain, France, Italy, Greece, the Central and Eastern European countries Bulgaria, Romania, Macedonia, Serbia, Croatia, Hungary, Ukraine and Russia.  In the UK, the probability of sustained *P falciparum* transmission by 2100 is small, even under the most extreme scenarios; however, a *P vivax* model predicts increasing climatic suitability in southern Great Britain by 2030; by 2080, southern Scotland will be climatically suitable for malaria transmission.  In China, increases in temperature, relative humidity and sunshine hours led to an increase in monthly malaria cases; it is predicted that if there are no preventive measures, the geographical scope of certain malaria vectors such as Anopheles minimus and Anopheles dirus and accordingly, the incidence of malaria may increase by 2030.  Projections show a reduction in the climate suitability for Anopheles in the Philippines in 2100 because of changes in heat stress, causing large areas to have heat stress beyond the maximum survival values for the malaria vector (>40C).  In Nepal, the distribution of malaria, which was previously confined to lowland valleys and hills (<1,200 m altitude) is now observed to extend to the hills and mountains (> 2,000 m) causing seasonal malaria epidemics; while the short-term data shows a clear association between climatic factors and VBDs, but it is complex and difficult to project long-term effects of climate change in the face of rapid environmental and socio-economic changes and attribution to climate change remains unclear. | Bardosh et al., 2017  Cella et al., 2019, Swynghedauw, 2009, Zhang et al., 2008  Watts et al., 2020, Yu et al., 2015  Giesen et al., 2020  Yu et al., 2015  Fischer et al., 2020  Medlock and Leach, 2015  Yi et al., 2019  Chua et al., 2019  Dhimal et al., 2015 |
| Adaptation Strategies  Enhanced surveillance | 2 | Community-based adaptation strategies include vector surveillance and risk mapping.  In the UK, surveillance of endemic and non-native vectors is being done through passive vector surveillance schemes | Bardosh et al., 2017  Medlock and Leach, 2015 |
| Early warning systems for outbreak detection | 3 | In South Africa, systems for forecasting extreme weather, and tracking malaria to support malaria programming appear well established, e.g. the Infectious Diseases Early Warning System project (iDEWS) project can forecast changes in malaria incidence and the geographical expansion of disease outbreaks.  Different malaria epidemic detection algorithms have been developed; these yielded different results in different settings. There is evidence that the source, type and quality of malaria data used have an impact on the reliability of the analysis or forecast depending on the aspect of the Malaria Early Warning System (MEWS) being modelled.  In the Philippines, adaptation strategies included associating climate/weather variables and health outcomes by using advanced modelling techniques and predictive models as part of an early warning system for disease prevention and control programs. | Chersich and Wright, 2019  Mabaso and Ndlovu, 2012  Chua et al., 2019 |
| Predictive models of future climate change impacts | 1 | In the UK, improved models of future climate change impacts should incorporate the many drivers for change (including climate) for a range of vector-borne diseases; in addition to climate-based models, vector models should focus on land-use and its change and host distributions and abundance. | Medlock and Leach, 2015 |
| Enhanced vector control | 2 | In the UK, targeted wetland management approaches might be an option to control mosquito habitats; new habitats designed as part of climate-change adaptation (to address flooding and mitigate storm surges) should be assessed in terms of environmental effect and future disease risk from mosquitoes and should include plans to mitigate nuisance or vector mosquitoes.  Community-based adaptation strategies include those that address housing and the domestic environment, modifying natural environments, animal-based interventions, water, sanitation and hygiene (WASH), chemical vector control, and access to biomedical interventions. | Medlock and Leach, 2015  Bardosh et al., 2017 |
| Health systems strengthening | 1 | The core capacities of countries’ health-care systems, as outlined by WHO’s International Health Regulations, have been shown to be effective predictors of protection against disease outbreak, with further data required to confirm any long-term shift. | Watts et al., 2021 |
| Dengue | **No. articles** | **Results** | **Citations** |
| Climate Impacts  Climate/weather-disease associations | 13 | In South America, there was a positive association between precipitation, temperature and humidity and dengue incidence, and the presence of larvae of A. aegypti with the El Niño phenomenon.  In Peurto Rico, there was a positive association between monthly variations in temperature, precipitation, and dengue incidence. The effect of temperature on dengue incidence was highest in Puerto Rico’s mountainous area, while the effect of precipitation was greatest in the southwestern coastal region.  In Europe, temperature seems to be the most important factor for the establishment and dynamics of *Ae. albopictus*. Winter temperatures limit its distribution range to areas with temperatures above 0 C.  In Pakistan, the highest dengue cases occur during July–September with more rainfall, optimum temperature and humidity, providing a conducive environment for breeding, survival and growth of Aedes mosquitoes.  In China, temperature, precipitation, humidity and air pressure were considered as major weather factors for dengue fever transmission; the climate-DF association depends largely on local climate conditions. Ae. albopictus have extended their geographic range to with an annual mean temperature below 11°C and a January mean temperature below -5°C highlighting that most provinces and cities in China now at risk for dengue.  In the Asia-Pacific region, dengue outbreaks coincide with wet season; positive associations between dengue incidence and precipitation were reported in many countries; spatial variation in the ENSO–dengue relationship was observed across countries.  In Nepal, DENV and its vector Ae. aegypti have expanded across the country in conjunction with pronounced warming in the mountains regions 2,000 m above sea level.  In Malaysia, several studies found that temperature and rainfall significantly influenced dengue trends at different lag periods; the highest risk of dengue cases was observed from 21 to 26 °C at a lag of 1-8 weeks. | López et al., 2018  Matysiak and Roess, 2017  Brugueras et al., 2020  Khan et al., 2018, Ahmed et al., 2016 & 2019  Li et al., 2018, Bai et al., 2013, Yi et al., 2019  Filho et al., 2019, Banu et al., 2011  Dhimal et al., 2015  Hii et al., 2016 |
| Climate change impacts on disease risk | 8 | Dengue prevalence will increase due to the effects of CC in several African countries in Southern and Central Africa.  In South America, an extension of the latitudinal range of dispersion of Aedes aegypti is expected with spread from tropical zones to temperate zones.  In Puerto Rico the incidence of dengue has been relatively constant over time, despite global warming, possibly due to declining rainfall, improving health care and minimal changes in population size.  In Europe, studies suggest a possible increase in epidemic potential with a south to north gradient by the end of 21st century, with an increase in climatically suitable areas for the establishment of *Ae. albopictus* in western, central and eastern Europe, and a decline in southern Europe by 2050 and until 2100. An increasing risk for DENV is predicted, especially around the Mediterranean and Adriatic coasts, towards the end of 21st century.  Climate change assessments predict that southeast England will become more suitable for the establishment of *Ae. albopictus, while c*olonization by *Ae. aegypti* is not expected to become established up to 2100.  In China, projections of DF using climate models suggest an increase in the number of days per year and total area conducive to DF transmission due to climate change.  From 1950 to 2018, the global climate suitability for the transmission of dengue increased by 8·9% for Aedes aegypti and 15·0% for Aedes albopictus. 2018 was particularly favourable for the transmission of dengue, with a global rise in vectorial capacity of 8·9% for A aegypti and 15·0% for A albopictus compared with a 1950s baseline. Although average suitability for dengue remained low in Europe, 2018 was the most suitable year yet recorded for both vector species in this region, with a change from the 1950s baseline of 25·8% for A aegypti and 40·7% for A albopictus.  The transmission of dengue is highly sensitive to climatic condition; minimum, maximum and mean temperatures, relative humidity and rainfall were the most important climate variables that predict dengue incidence, however, these variables are predictive at specific lags of time. | Giesen et al., 2020  López et al., 2018  Matysiak and Roess, 2017  Brugueras et al., 2020  Medlock and Leach, 2015  Li et al., 2018  Watts et al., 2020  Naish et al., 2014 |
| Adaptation Strategies  Enhanced surveillance | 2 | In the UK, surveillance of endemic and non-native vectors is being done through passive vector surveillance schemes, and will require maintenance and expansion of existing trap networks across the UK, and targeted surveillance for the incursion of non-native invasive vector species.  Community-based adaptation activities include vector surveillance and risk mapping. | Medlock and Leach, 2015  Bardosh et al., 2017 |
| Early warning systems for outbreak detection | 2 | In the UK, it will be necessary to work with environmental organizations to develop vector management plans and forecasting methods to prepare for a disease outbreak resulting from an extreme event.  In the Philippines, adaptation strategies included associating climate/weather variables and health outcomes by using advanced modelling techniques and predictive models as part of an early warning system for disease prevention and control programmes; model results suggest the need intensified vector control for dengue, especially during the rainy season or months with high rainfall. | Medlock and Leach, 2015  Chua et al., 2019 |
| Predictive models of future climate change impacts | 1 | In the UK, improved models of future climate change impacts should incorporate the many drivers for change (including climate) for a range of vector-borne diseases; in addition to climate-based models, vector models should focus on land-use and its change and host distributions and abundance. | Medlock and Leach, 2015 |
| Enhanced vector control | 2 | The World Mosquito Program in the Pacific region identified a promising approach to Dengue and other Aedes-borne disease control using the bacterium Wolbachia  Several community-based adaptation strategies exist to reduce vector-borne disease transmission and risk, including those that address housing and the domestic environment, modifying natural environments, Water, sanitation and hygiene (WASH), and chemical vector control. | Filho et al., 2019  Bardosh et al., 2017 |
| Health systems strengthening | 1 | The Nepal National Adaptation Programme of Action (NAPA) to climate change has identified vector-borne diseases as one of the highest priority adaptation projects for the health sector in Nepal; measures include development of robust surveillance systems (health and vector surveillance); increase of diagnostic capacities and training; implementation of vector control measures, including individual and collective protection; and increased awareness and community engagement concerning mosquito-borne diseases. | Dhimal et al., 2015 |

**Table S2:** Search strategies for the electronic databases used to identify review articles

| Database | Search terms |
| --- | --- |
| PubMed | ("climate change"[Title/Abstract] OR "greenhouse effect"[Title/Abstract] OR "changing climate"[Title/Abstract] OR "global warming"[Title/Abstract] OR "extreme weather"[Title/Abstract] OR "climate variability" OR "greenhouse gas" OR "rising temperature" [Title/Abstract]) AND (("meta analysis"[Publication Type] OR "review"[Publication Type] OR "systematic review"[Filter]) AND 2007/01/01:2020/12/31[Date - Publication]) AND (("malaria"[Title/Abstract] OR "dengue"[Title/Abstract]) AND (("meta analysis"[Publication Type] OR "review"[Publication Type] OR "systematic review"[Filter]) AND 2007/01/01:2020/12/31[Date - Publication])) |
| Scopus | ( TITLE-ABS-KEY ( ( "Climate Change" OR "greenhouse effect" OR "changing climate" OR "global warming" OR "extreme weather" OR "climate variability" OR "greenhouse gas" OR "rising temperature" ) ) ) AND ( TITLE-ABS-KEY ( ( malaria OR dengue) ) ) AND ( LIMIT-TO ( DOCTYPE , "re" ) ) |
| Epistemonikos | (title:((“climate Change" OR "greenhouse effect" OR "changing climate" OR  "global warming" OR "extreme weather" OR "climate variability" OR "greenhouse gas" OR "rising temperature") AND (malaria OR dengue)) OR abstract:((“climate Change" OR "greenhouse effect" OR "changing climate" OR "global warming" OR "extreme weather" OR "climate variability" OR "greenhouse gas" OR "rising temperature") AND (malaria OR dengue))) |
